# Arousal and attention re-orienting in autism spectrum disorders: evidence from auditory event-related potentials

**DOI:** 10.3389/fnhum.2014.00034

**Published:** 2014-02-06

**Authors:** Elena V. Orekhova, Tatiana A. Stroganova

**Affiliations:** ^1^MEG Centre, Moscow State University of Psychology and EducationMoscow, Russia; ^2^MedTech West, Sahlgrenska AcademyGothenburg, Sweden

**Keywords:** autism spectrum disorders (ASD), arousal, attention re-orienting, sensory modulation, auditory event-related potentials, cholinergic pathways, nicotine

## Abstract

The extended phenotype of autism spectrum disorders (ASD) includes a combination of arousal regulation problems, sensory modulation difficulties, and attention re-orienting deficit. A slow and inefficient re-orienting to stimuli that appear outside of the attended sensory stream is thought to be especially detrimental for social functioning. Event-related potentials (ERPs) and magnetic fields (ERFs) may help to reveal which processing stages underlying brain response to unattended but salient sensory event are affected in individuals with ASD. Previous research focusing on two sequential stages of the brain response—automatic detection of physical changes in auditory stream, indexed by mismatch negativity (MMN), and evaluation of stimulus novelty, indexed by P3a component,—found in individuals with ASD either increased, decreased, or normal processing of deviance and novelty. The review examines these apparently conflicting results, notes gaps in previous findings, and suggests a potentially unifying hypothesis relating the dampened responses to unattended sensory events to the deficit in rapid arousal process. Specifically, “sensory gating” studies focused on pre-attentive arousal consistently demonstrated that brain response to unattended and temporally novel sound in ASD is already affected at around 100 ms after stimulus onset. We hypothesize that abnormalities in nicotinic cholinergic arousal pathways, previously reported in individuals with ASD, may contribute to these ERP/ERF aberrations and result in attention re-orienting deficit. Such cholinergic dysfunction may be present in individuals with ASD early in life and can influence both sensory processing and attention re-orienting behavior. Identification of early neurophysiological biomarkers for cholinergic deficit would help to detect infants “at risk” who can potentially benefit from particular types of therapies or interventions.

## Introduction

Autism spectrum disorders (ASD) are neurodevelopmental disorders that are primarily characterized by impairments in social and communication skills and by repetitive and stereotypical behaviors. Apart from the “core” deficits in social and communication domains, people with ASD show relatively low-level abnormalities such as difficulties with regulating arousal level, problems with modulating sensory input, and atypical attention. Depending on the context, attention of individuals with autism may be either overtly over-focused or have no direction whatsoever (Hermelin and O'Connor, 1970; Allen and Courchesne, 2001). Furthermore, they may demonstrate either abnormally elevated or unusually low level of behavioral and autonomic arousal (Kinsbourne, 1987; Hirstein et al., 2001). The other feature of ASD is a frequent occurrence of sensory modulation difficulties manifested in either hyper- or hypo-responsiveness to sensory stimuli (Grandin and Scariano, 1996; Ben-Sasson et al., 2008). The sensory modulation difficulties and attention problems in ASD were observed in different sensory modalities and occur despite the lack of any apparent deficits of the corresponding sensory function.

The role of “low-level” abnormalities of arousal, attention orienting, and sensory responsiveness in people with ASD has been discussed since it had been established by the scientific community that autism has a biological, rather than a social underpinning (Rimland, 1964; Ornitz and Ritvo, 1968; Cohen et al., 1976; Ornitz, 1989). Gradually, the focus of autism research has shifted to investigating higher-order disturbances, such as the “theory of mind” deficit (Baron-Cohen et al., 1985), decreased central coherence (Frith and Happe, 1994), and impaired “mirror neurons” functioning (Williams et al., 2001). Arousal and attention abnormalities are again coming into the focus of autism research as it has become evident that they may precede social symptoms and represent the earliest signs of autism in infants at risk for ASD (Zwaigenbaum et al., 2005; Elison et al., 2013; Elsabbagh et al., 2013).

Co-occurrence of arousal abnormalities, atypical attention, and sensory modulation problems in the same individuals with ASD encouraged researchers to look for causal links between these phenomena. These links are, however, far from clear. Ben-Sasson et al. (2008) suggested that co-occurrence of extreme under- and over-reactive (avoiding) behaviors in children with autism may result from abnormal arousal regulation. Liss et al. (2006) and Allen and Courchesne (2001) proposed that both behavioral under- and over-reactivity may be a matter of the individual's attention distribution. Liss et al. (2006) suggested that over-focused attentional style in ASD may be the result of hyperarousal, while Keehn et al. (2013) hypothesized that atypical behavioral arousal regulation in persons with ASD results from early deficits in disengaging attention.

Obviously, it is difficult to draw firm conclusions about the roles of attention and arousal disturbances in ASD neuropsychopathology without looking at the underlying physiological mechanisms. Since basic sensory processes appear to be generally intact in people with ASD, their abnormally reduced or exaggerated responses to environmental stimuli may reflect modulatory influences on information processing in cortical networks. One way to study these modulatory mechanisms would be to look at brain responses to sudden changes in outer environment. The event-related potentials (ERP) and magnetic fields (ERF) have perfect time resolution and may help to investigate neuro-functional deficits in ASD by pinpointing the affected processing stages. In the present paper, we summarize current evidence on arousal and attention orienting abnormalities in ASD within behavioral domain and then discuss studies that applied ERP and ERF techniques to explore relevant physiological deficits. These will include “sensory-gating-type” studies that analyzed “obligatory” auditory ERP components in order to look at pre-attentive phasic arousal as well as research on two further stages of processing—automatic detection of physical changes in auditory stream indexed by mismatch negativity (MMN) and evaluation of stimulus novelty indexed by the P3a component. Taking into account that the majority of sensory gating, MMN, and P3a studies in ASD used auditory stimuli, we will limit our discussion to the auditory modality. As a whole, the auditory ERP/ERF studies suggest that processing of attended auditory stimuli is either normal or increased in individuals with ASD, while processing of unattended stimuli is usually decreased. We speculate that this is a result of a deficit in the relatively early, pre-attentive arousal processes and that the right hemisphere of the brain may play a particular role in this deficit. Although the pre-attentive arousal deficit was documented in the auditory domain, the supramodal nature of attention and sensory modulation problems in ASD (Murray et al., 2005; Leekam et al., 2007; Bonneh et al., 2008) infers that abnormal pre-attentive arousal is present across sensory modalities.

In conclusion, we will speculate that the difficulties in attention re-orienting and sensory modulation as well as abnormal physiological arousal are related to a deficit in the nicotinic branch of the cholinergic arousal system that is well documented in ASD (Deutsch et al., 2010; Anand et al., 2011). Our focus on the cholinergic system is explained by its commonly acknowledged role in attention re-orienting and arousal (Everitt and Robbins, 1997; Robbins, 1997; Sarter and Bruno, 2000; Greenwood et al., 2005, 2009, 2012; Sarter et al., 2005; White and Yee, 2006; Giessing et al., 2012). Apart from cholinergic abnormalities, individuals with ASD demonstrate prominent abnormalities of GABA- and glutamatergic neurotransmission (Oblak et al., 2010, 2011; Fatemi et al., 2013). There are complex mutual interactions between these neurotransmitters and cholinergic pathways (see e.g., Albuquerque et al., 2009) that are to be addressed by the future neural modeling studies of ASD.

## Difficulties with regulating arousal, attention, and sensory processing in ASD

### Arousal

The term “arousal” was originally used to describe both behavior and physiological activity, including its cortical and autonomic components (Lacey, 1967). Arousal can be subdivided into tonic and phasic. The tonic arousal describes relatively slow fluctuations in the energetic arousal state during sleep and wakefulness, while phasic arousal responses indicate the organism's energetic reaction to specific stimulus events (Combs and Polich, 2006). Tonic and phasic arousal are interdependent and produce strongly overlapping activation of a predominantly rightward-lateralized frontal, parietal, thalamic, and brain-stem network (Sturm and Willmes, 2001).

Fluctuations in arousal level during sleep and wakefulness are mediated by multiple arousal systems of the brain (Robbins, 1997; Dringenberg and Vanderwolf, 1998; de Lecea et al., 2012). These systems differ in respect to the primary neurotransmitter, are characterized by some specificity of cortical and subcortical projections, and have different functions in arousal-like processes (Robbins, 1997). The coeruleo-cortical noradrenalin (NA) system mediates brain capacity to maintain “alerting” to salient external stimuli. The mesolimbic and mesocortical dopamine (DA) systems play a role in the activation of output, whether cognitive or motor in nature, and are critically important for executive functions. The cholinergic (ACh) system mediates regulation and allocation of processing resources as well as attention and memory processes and is involved in attention re-orienting in space (Witte et al., 1997; Giessing et al., 2012; Greenwood et al., 2012). The serotonergic (5-HT) system serves to dampen the actions of all other systems by promoting behavioral inhibition and cortical de-arousal. Different arousal systems interact closely. For example, the classically recognized cortical activating effects of ascending NA system depends, to a significant extent, on a basal forebrain cholinergic input to the cortex (Dringenberg and Vanderwolf, 1998; Berntson et al., 2003).

Thus, arousal is a complex phenomenon and different neuro-functional abnormalities may contribute to its dysregulation in ASD. There are strong arguments for the presence of abnormalities of cholinergic (Deutsch et al., 2010; Anand et al., 2011) and serotoninergic (Harrington et al., 2013) systems, while the evidence for the role of dopaminergic and noradrenergic systems is less conclusive (Canitano and Scandurra, 2011).

Various aspects of behavioral and autonomic indicators of arousal are affected in ASD. Sleep disturbances are commonly observed in children with ASD and correlate with autism severity (Tudor et al., 2013). Tonic arousal, understood as a degree of vigilance or alertness during wakefulness, was suggested to be either abnormally increased (Hutt et al., 1964, 1965), decreased (Rimland, 1964), or unstable, possibly due to excessive or fluctuating ascending activation in the brain (Hermelin and O'Connor, 1970; Kinsbourne, 1987). Phasic arousal is also atypical in people with ASD, with either decreased or increased reactions to stimuli (van Engeland, 1984; Hirstein et al., 2001; Schoen et al., 2008).

The most widely used measures of autonomic arousal are the tonic skin conductance level (SCL) and spontaneous and stimulus–related fluctuations in electro-dermal activity (EDA). Schoen et al. (2008) have found that some children with autism had high SCL (high tonic arousal) associated with higher than normal EDA magnitudes, faster latencies, and slower habituation; while others had low SCL (low tonic arousal) linked with lower EDA magnitudes, slower latencies, and faster habituation. The authors concluded that children with autism represent a heterogeneous population in terms of autonomic arousal.

One factor that contributes to the large inter-individual variability of SCL and EDA in individuals with ASD may be the excessive context-dependent fluctuation of these measures. In study of Hirstein et al. (2001) children with autism mainly had higher than normal baseline SCL and high-amplitude EDA. In the majority of the children, however, SCL and EDA dropped below the values observed in normal control groups as soon as they became involved in self-stimulatory activities (provoked by putting their hands in a bowl with beans).

van Engeland (1984) have noted that children with autism were often electrodermally *nonresponsive* to the first stimulus in a row of sounds, thus resembling schizophrenic patients. As such abnormal non-responsiveness was observed only to the first stimulus in a row, the authors concluded that it might be a state-dependent phenomenon, possibly reflecting abnormal allocation of attention.

Difficulties with arousal regulation may be present in children with ASD very early in life, well before the social symptoms of autism become evident. The incidence of sleep problems during the first 2 years of life is higher in children with ASD than in either typically developing children or those with mental retardation without autism (Dahlgren and Gillberg, 1989). Increased irritability and proneness to distress have been observed in toddlers later diagnosed with ASD (Bryson et al., 2007).

Interestingly, indications of atypical arousal regulation have been found even in 4 months-old graduates of neonatal care unit (ICU), who later went on to ASD (Karmel et al., 2010; Cohen et al., 2013). These infants had an abnormally high preference for more arousing visual stimulation, which reliably differentiated them from the ICU graduates without ASD, irrespective degree of their CNS injury. Moreover, in participants without significant brain damage the preference for higher rates of stimulation at 4 months correlated with lower social competence at 3 years, but only in those with initially abnormal auditory brain-stem potentials (Cohen et al., 2013). This combination of brain stem and arousal abnormalities suggests that damage to the brain stem that leads to arousal dysregulation is a risk factor for autism.

To sum up, atypical arousal and/or problems with arousal regulation are already present in individuals with ASD in their infancy. In older children, the predominant type of arousal abnormality may vary between different individuals, as well as within the same individual, and depends on this individual's immediate state. Arousal dysregulation manifests itself in two distinct modes of functioning. The first mode is characterized by elevated tonic arousal, anxiety, and difficulties to concentrate attention. The second mode is reflected in reduced tonic arousal, self-stimulatory activities, and decreased awareness of surrounding outside of the current attention focus.

### Attention

Attention abnormalities in children with autism have been already described by Leo Kanner, who observed not only a lack of attention to people in these pediatric patients, but also admitted that many of them were “oblivious to all but immediate focus of attention” (Kanner, 1968). Hermelin and O'Connor (1970) have noted that attention in autistic individuals is either overtly over-focused or has no direction whatsoever. Since these early descriptive studies, the narrow “spot-light” focus of attention and difficulties with re-directing attention were repeatedly reported in scientific literature (Kinsbourne, 1991; Allen and Courchesne, 2001; Ames and Fletcher-Watson, 2010) as well as in personal accounts of individuals with autism (Grandin and Scariano, 1996). Importantly, problems with re-directing attention in autism are evident in both social and nonsocial domains (Townsend et al., 1996; Dawson et al., 1998; Harris et al., 1999; Zwaigenbaum et al., 2005; Baranek et al., 2013), suggesting a generalized deficit in re-orienting. Moreover, these attention difficulties are rather specific for autism, as they discriminate children with autism not only from typically developing children but also from children with developmental delay without autism (Dawson et al., 1998).

Different psychophysical paradigms have been applied to elucidate the nature of attention abnormalities in ASD (see Ames and Fletcher-Watson, 2010 for review). Many studies used modifications of an experimental paradigm developed by Posner et al. (1984). According to Posner, attention can be subdivided into three relatively independent modules related to alerting, orienting, and executive control. Posner's paradigm is applicable to measuring alerting and orienting modules. The orienting module includes processes of disengagement, shifting, and reengagement. Participants are usually presented with visual stimuli appearing on a computer screen to their left or to their right and asked to press a button in response to the “target” stimulus. The target is usually preceded by a warning visual signal (i.e., “cue”). Spatial position of the cue relative to the target as well as time interval between the cue and the target are manipulated across the paradigms. There are usually 4 types of spatial “cue and target” combinations: no cue, spatially non-informative, congruent, and non-congruent. In the no-cue condition, the target is not preceded by a cue altogether. Spatially non-informative or neutral cues may be bilateral or appear in the center of the screen and provide the subject with a non-spatial alert of a coming target. Congruent or valid cues are presented in the same place where the target will appear. Finally, non-congruent or invalid cues are shown in the location opposite to the target. Neutral cues alert the subjects and decrease reaction time to the target relative to the “no-cue” condition. Fastest responses are observed in case of validly cued targets, because attentional resources are directed to the cued location in advance of the target appearance. Invalid cue, on the other hand, prolongs subject's reaction time because attention has to be disengaged and re-oriented from the invalidly cued location to a validly cued one. Thus, effectiveness of different attention orienting processes can be measured by comparing subjects' reaction times between the four cue-target combinations. For example, alerting is measured by the difference in reaction time between “no-cue” and “neutral cue” conditions. The orienting-shifting score can be assessed by comparing reaction time between “neutral” and “congruent” cues. Finally, attention disengagement efficacy is measured by comparing reaction time between “congruent or valid” and “non-congruent or invalid” conditions.

The other task that is often used to measure attention disengagement is the “gap-overlap” task. In the “gap” condition, the central fixation stimulus disappears before presentation of the peripheral target stimulus. In the “overlap” condition, the central fixation stimulus remains on the screen during target presentation. In order to execute saccade to the peripheral target under the “overlap” condition, participants have to disengage attention from the central stimulus, while such disengagement is not necessary in the “gap” condition. In this case disengagement is measured as a difference in saccade latency between overlap and gap conditions.

Both Posner and “gap-overlap” paradigms were applied to evaluate alerting and orienting in ASD. No apparent abnormalities in alerting have been found in people with ASD using a modification of Posner paradigm (Keehn et al., 2010). Some studies have found that children with ASD were either generally slower to shift attention (Keehn et al., 2010) or performed less number of rapid attention shifts (Landry and Bryson, 2004) as compared to the typically developing children. Others reported in participants with ASD greater than normal number of “express” saccades with extremely short reaction time to the target stimuli during the “overlap” condition in the “gap-overlap” task (Kawakubo et al., 2004), or demonstrated less than normal differences in saccade latency between the gap and overlap conditions (van der Geest et al., 2001). These latter findings are indicative of engagement rather than disengagement problems in ASD.

Since disengagement is a function of initial engagement, it can be altered through the manipulation of stimuli (Marshall, 2011). A certain level of initial engagement by (i.e., interest to) the central fixation stimulus is, therefore, necessary in order to reveal the disengagement deficit in ASD. The studies that probably fulfilled this requirement did find attention disengagement problems in ASD using gap-overlap tasks (Landry and Bryson, 2004; Kawakubo et al., 2007; Elsabbagh et al., 2013).

The majority of studies that used Posner's-type paradigms also reported the disengagement deficit in people with ASD (Casey et al., 1993; Wainwright-Sharp and Bryson, 1993; Townsend et al., 1996; Harris et al., 1999; Renner et al., 2006). Importantly, in order to reveal this disengagement deficit, the delay between the cue and the following target stimulus in the Posner's paradigm had to be long enough to allow for successful *engagement* of attention by the preceding cue (Wainwright-Sharp and Bryson, 1993). Generally, the slow disengagement of attention is one of the most robust attention deficits found in ASD (Ames and Fletcher-Watson, 2010).

Remarkably, problems with shifting attention and, particularly, with disengagement of attention were found even in infants at high risk for autism (Zwaigenbaum et al., 2005; Elison et al., 2013; Elsabbagh et al., 2013) and predicted later diagnosis. It has been widely discussed in the literature that deficits in early-developing attentional systems have a profound effect on the long-term prognosis of a child with autism. General impairments in orienting to social as well as nonsocial sensory stimuli may cause a cascade of developmental consequences for later-developing social communicative functions, including joint attention and language development (Mundy and Neal, 2001; Mundy and Jarrold, 2010; Baranek et al., 2013). In line with this hypothesis, a recent study has shown that impairments in attention disengagement in children with ASD correlate with higher severity of core autism symptoms (Bahrick and Todd, 2013).

The term “disengagement” introduced by Posner et al. (1984) closely overlaps with the term “re-orienting” used by Corbetta et al. (2008). Corbetta and colleagues have delved more closely into activity of neural networks responsible for automatic shifts of attention to unattended but salient targets. On the cortical level, the system subserving re-orienting/disengagement includes temporo-parietal junction (TPJ) and ventral frontal cortex (VFC), which in turn is comprised of certain parts of middle frontal gyrus, inferior frontal gyrus, frontal operculum, and anterior insula (Corbetta et al., 2008; Corbetta and Shulman, 2011). These cortical areas in combination with subcortical arousal systems constitute a strongly rightward-lateralized ventral attentional network (VAN).

At the neural level, problems with attention disengagement/re-orienting in ASD are likely to relate to a failure of the VAN, a deficiency at subcortical levels preceding VAN activation or a break in communication between the two. Taking into account that VAN strongly overlaps with cortical areas involved in social cognition (Corbetta et al., 2008), its dysfunction in ASD would also be in line with a co-occurrence of attention orienting and social deficits in these disorders.

It should be noted, however, that problems with attention orienting are not unique for autism, but were also observed in young people with Williams syndrome who do not display the joint attention or social deficits associated with ASD (Lincoln et al., 2002). Results of Lincoln et al. (2002) warn that attention re-orienting/disengagement deficit in early development is, by itself, not sufficient to cause later social impairments and that specific neural factors underlying the attention disengagement abnormalities in ASD may play the key role.

To sum up, the reduced ability to disengage (a previously engaged) attention is one of the most consistently found cognitive deficits in individuals with ASD from infancy onwards. Notably, this behavioral deficit is not specific to social stimuli, suggesting a general failure of attention networks. Such failure appearing early in life may have an important contribution to abnormal development of social cognition.

### Modulation of sensory input

High incidence of sensory modulation problems, including both hyper- and hypo-responsiveness to stimuli of different modalities is observed in individuals with ASD across their life span (O'Neill and Jones, 1997; Harrison and Hare, 2004; Crane et al., 2009; Wiggins et al., 2009) and differentiates them from both neurotypical individuals and those with developmental delay. The importance of abnormal sensory sensitivity in ASD neuro-phenotype has been recently recognized by including it into the new edition of the Diagnostic and Statistical Manual of Mental Disorders (DSM-5) as a group of clinically relevant symptoms. The two sensory response patterns (hyper-responsiveness and hypo-responsiveness) may coexist in the same individual and be expressed in a context-dependent manner (Hirstein et al., 2001; Baranek et al., 2006). The co-occurrence of extreme under- and over-reactive behaviors in children with ASD may indicate a common etiology underpinning poor sensory modulation, such as abnormal attention (Liss et al., 2006) or arousal regulation (Ben-Sasson et al., 2008).

It has been suggested that *non-responsiveness* to sensory stimuli may particularly strongly contribute to development of autism via an interruption of basic orienting responses that are foundational for the development of joint attention skills (Baranek et al., 2013). Liss et al. (2006) analyzed co-occurrence of sensory and attention disturbances (over-focusing) in a big sample of children with ASD and have found that subjects characterized by over-focused attention style were also characterized by high over- and under-reactivity and had the most severe symptoms of autism, although not necessarily lower IQ. In a recent study, Baranek et al. (2013) have shown that low sensory responsiveness to both social and non-social stimuli in young children with ASD was associated with low mental age and predicted lower levels of joint attention and language. Watson et al. (2011) also reported in children with autism a significant association of *hypo*-responsiveness with the severity of social-communicative symptoms, while did not find such an association for *hyper*-responsiveness.

To sum up, co-occurence of sensory modulation difficulties, arousal regulation problems, and atypical attention in people with ASD suggests the presence of a common underlying physiological deficit. The non-responsiveness and failure to re-orient attention to novel or important (e.g., social) cues seem to be especially closely linked to the core features of autism. Below we review EEG and MEG studies that investigated different stages of information processing in individuals with ASD that are relevant for the understanding of their attention re-orienting problems.

## Processing of novelty and change in ASD: evidence from auditory ERP and ERF studies

Pre-attentive detection of stimulus salience or novelty is mandatory for automatic re-orienting of attention. In this section we will review ERP/ERF studies investigating pre-attentive and attention-related automatic processing of physically or temporally novel or deviant acoustic events in ASD. We will focus on the ERP/ERF components that are affected by stimulus novelty or change in stimuli stream. The earliest brain-derived auditory ERP component that displays these properties is P50 (Skinner et al., 2004; Nakagawa et al., 2013). We will start the discussion by looking at the latter ERP components (P3a and MMN), since these components have been the focus of the majority of the ERP/ERF studies in ASD.

### Automatic processing of novelty and change in the “oddball paradigm”

Attention re-orienting to novel stimulus is preceded by automatic detection of sensory change. Neural correlates of both change detection and attention re-orienting are often investigated using auditory oddball paradigm. In case of the “regular oddball,” the frequent “standard” stimuli are occasionally substituted by rare “deviants” that are different from the standard by pitch, duration, or other acoustic or semantic properties. Subjects either respond to the rare deviants (e.g., with a button press) or passively attend to the auditory stimulation stream. In the latter case they often watch an unrelated video presented in order to reduce boredom and/or distract attention from the auditory stimulation. Stimuli are usually presented with short (<1 s) intervals in order to preserve the echoic memory trace of the preceding sound and to facilitate automatic comparison.

MMN is a component of electrical/magnetic brain response elicited at 100–200 ms after the onset of change and is thought to signify a brain process responsible for change detection. It is measured as a difference wave between responses to deviant and standard stimuli. MMN is maximal over fronto-central scalp areas and is thought to be generated by the sources at superior temporal gyri (Jaaskelainen et al., 2004) and, possibly, also at frontal areas (Deouell, 2007). Large MMN amplitudes are associated with greater stimulus differences and accurate discrimination of these differences, suggesting that MMN is a cortical index of sound-discrimination accuracy (Naatanen et al., 2007).

P3a is associated with involuntary orienting of attention and is usually measured in a passive version of the oddball paradigm in response to unattended deviant stimuli. P3a is recorded over frontal and parietal cortical areas around 280 ms post-stimulus and is thought to be generated by distributed cortical sources comprising the attention re-orienting network (Mantini et al., 2009). “Novelty P3”—a component similar to P3a—is elicited in response to “novel” stimuli presented in a “novelty oddball” paradigm (Friedman et al., 2001). In this modification of the paradigm, P3 is measured in response to the highly deviant and/or unique novels presented among the more perceptually similar standards and deviants. Because the subject is not informed about these initially novel events, the “novelty oddball” procedure mimics more closely the real-world involuntary attentional capture by novel or unexpected events. Although novelty P3 and P3a are elicited by distinctly different stimuli during quite different task circumstances, they may reflect the output of a similar configuration of neural sources (Friedman et al., 2001). Both MMN and novelty-P3/P3a reflect functioning of neuronal mechanisms critically important for processing of novel or deviant events. MMN signifies initial detection of deviancy, whereas P3a is related to involuntary attention orienting and evaluation of those events for subsequent behavioral action (Friedman et al., 2001).

The majority of the MMN studies in ASD subjects used passive oddball paradigm while subjects *watched a silent movie* or read a book. The wide spectrum of obtained results probably reflects characteristics of the particular samples, stimulation parameters, and details of the experimental procedures. Many of the studies reported higher MMN amplitudes (Ferri et al., 2003; Lepisto et al., 2005, 2006, 2007; Korpilahti et al., 2007; Kujala et al., 2007, 2010) and/or shorted latencies (Gomot et al., 2002, 2011; Ferri et al., 2003; Lepisto et al., 2005; Korpilahti et al., 2007; Kujala et al., 2007) in individuals with ASD. These findings correspond well with behavioral data on superior auditory discrimination observed in these individuals (Mottron et al., 2000; Bonnel et al., 2003; O'Connor, 2012), their auditory hypersensitivity (Stiegler and Davis, 2010; Lucker, 2013), and their general hypersensitivity to change. Indeed, shorter MMN latencies in children with ASD correlated with a low tolerance of change (in place, time, people, food, and clothes) (Gomot et al., 2011). Some researchers, however, reported unremarkably normal MMN in ASD (Kemner et al., 1995; Ceponiene et al., 2003). Moreover, some EEG (Seri et al., 1999; Jansson-Verkasalo et al., 2003; Kujala et al., 2005, 2007; Lepisto et al., 2006; Andersson et al., 2013) and two available MEG studies (Tecchio et al., 2003; Cardy et al., 2005) have found decreased MMN amplitudes and/or prolonged MMN latencies in ASD participants. Interestingly, two studies investigating MMN to prosody deviants in Asperger's syndrome both reported significant but opposite findings. Children with Asperger's syndrome had higher MMN amplitudes and shorter latencies than the age-matched “neuro-typical” controls (Korpilahti et al., 2007), whereas adults with Asperger's syndrome had lower MMN amplitudes and longer latencies (Kujala et al., 2005).

Age, IQ, parameters of the stimulation (e.g., “speechness” of the sound), nature of stimulus change (e.g., pitch, intensity, duration)—all may have their roles in the difference of MMN findings reported by different authors. These roles are, however, far from being clear, because opposite MMN findings were reported for similar auditory stimuli and for groups of participants comparable in terms of their age and IQ. For example, *impaired* detection of deviance in ASD reflected by *reduced amplitude and/or increased latency* of MMN was observed in children (Seri et al., 1999; Jansson-Verkasalo et al., 2003; Cardy et al., 2005) and adolescents/adults (Tecchio et al., 2003; Kuhl et al., 2005; Andersson et al., 2013); in those with normal IQ (Jansson-Verkasalo et al., 2003; Lepisto et al., 2006; Andersson et al., 2013) and with mental retardation (Seri et al., 1999; Tecchio et al., 2003); in response to speech stimuli (Jansson-Verkasalo et al., 2003; Kujala et al., 2005) and in response to tones (Seri et al., 1999; Jansson-Verkasalo et al., 2003; Tecchio et al., 2003; Lepisto et al., 2006; Andersson et al., 2013). *Enhanced* detection of change in ASD reflected by *increased amplitude and/or shortened latency* of MMN has also been reported in children (Gomot et al., 2002, 2011; Ferri et al., 2003; Lepisto et al., 2005, 2006; Korpilahti et al., 2007; Kujala et al., 2010) and adults (Kujala et al., 2007; Lepisto et al., 2007); in those with normal or nearly-normal IQ (Lepisto et al., 2005, 2006, 2007; Korpilahti et al., 2007; Kujala et al., 2010) and with mental retardation (Ferri et al., 2003; Gomot et al., 2011); in response to speech (Korpilahti et al., 2007; Lepisto et al., 2007; Kujala et al., 2010) and non-speech (Gomot et al., 2002, 2011; Ferri et al., 2003; Lepisto et al., 2005; Kujala et al., 2007) stimuli.

We propose that such variability in findings might, at least partly, reflects the degree of subject's attentiveness to the stream of the test stimuli vs. his/her attention to the surroundings (e.g., to a movie used to keep the subject busy during the auditory experiment) or even to the subject's internal feelings or thoughts. In its turn, direction of attention during passive oddball paradigm could be potentially modulated by a number of factors, such as attractiveness of the movie for the particular age/IQ, quality of the auditory test stimuli (e.g., speech, non-speech), the level of anxiety in unfamiliar experimental settings, etc.

Although MMN is believed to be relatively insensitive to the direction of subject's attention, as it is present even in sleep (Ibanez et al., 2009), the direction of attention to/away from the auditory stimulation does influence MMN amplitude in healthy children (Gomes et al., 2000). Moreover, amplitudes of the auditory MMN and P3a are modulated by the difficulty of the interfering visual task (Yucel et al., 2005; Rissling et al., 2013), thus reflecting the impact of available attention resources on physiological processes related to automatic change detection or involuntary attention switching. Considering limited attention capacities (narrow attention focus, difficulties with attention re-orienting) in people with ASD, direction of their attention to the auditory stream may have a disproportionately large role for the MMN and the P3a responses when compared to neurotypical individuals. For example, the presence of an interfering auditory perceptual stream (e.g., a movie with a sound track, irrelevant to the auditory oddball stimulation) may deteriorate processing of test auditory stimuli in children with ASD to a stronger degree than in children without ASD.

Indeed, two studies applying sound stimuli over an acoustic background of a simultaneously presented movie, have found a strongly reduced MMN in response to either tone (Dunn et al., 2008) or speech (Kuhl et al., 2005) stimuli in children with ASD. These finding are especially convincing taking into account quite representative samples of these studies (Dunn et al., 2008: 34 ASD and 34 control subjects; Kuhl et al., 2005: 29 ASD and 15 controls). Dunn with coauthors repeated the study with a smaller sample of participants using both “passive/sound track” and “active” oddball conditions. The latter task modification required subjects' attention to the test stimuli and their manual response. In this second experiment, participants with ASD displayed normal MMN in the “active” condition, but, again, lacked MMN in the “passive/sound track” condition, thus supporting the role of limited attention recourses in MMN generation in ASD.

If MMN is evoked by salient stimulation, it is normally followed by a P3a wave. Similar to the MMN results, the P3a findings in ASD also range from virtual absence to increased amplitude and shorter latency. The early studies have shown that P3a is strongly reduced in children with ASD in response to task-irrelevant highly deviant novel sounds (non-speech noises) in the “novelty oddball” paradigm (Courchesne et al., 1984; Kemner et al., 1995). Courchesne et al. (1984, 1985) have also found in individuals with ASD decreased amplitude of the anterior negative component following P3a to “novels” and peaking at Cz with latency between 600 and 1000 ms (i.e., A/NCz/800). This component resembles re-orienting negativity later described by Schröger and Wolff (1998) and may be linked to re-orienting from task-irrelevant “novels” toward task-relevant aspects of stimulation. Therefore, similarly to the reduced P3a, the reduced A/NCz/800 might reflect diminished processing of unattended novel sounds in ASD.

The studies mentioned above employed an active oddball task, where subjects were required either to push a button in response to the deviant stimuli (Courchesne et al., 1984) or to count them (Kemner et al., 1995). Similarly to the previous MMN findings (Kuhl et al., 2005; Dunn et al., 2008), the abnormally deceased P3a response to the task-irrelevant “novel” sounds observed in individuals with autism in these studies may reflect their limited attention resources, rather than a failure to process novelty. Specifically, the presence of a task might facilitate linking of sequential sound elements together into a segregated auditory stream of “standards and deviants” thus placing a *unique* novel sound outside of this attended stream. Therefore, while focusing on a particular task-relevant stimulation, people with ASD may fail to detect even salient acoustic events that do not belong to this attended stream.

The majority of other studies investigating P3a in ASD used a passive ‘regular oddball’ paradigm. Among these, the studies that used speech stimuli often reported decreased P3a amplitudes (Ceponiene et al., 2003; Lepisto et al., 2005, 2006, 2007), while those applying non-speech sounds reported increased P3a amplitudes and/or shortened latencies (Gomot et al., 2002, 2011; Kujala et al., 2007; Lepisto et al., 2007). Some researchers have suggested that the orienting deficit reflected in P3a reduction in autism might be speech–sound specific (Ceponiene et al., 2003). Whitehouse and Bishop (2008), however, have shown that reduced P3a in response to speech deviants in individuals with ASD can be better explained by their generally reduced attentiveness to the stream of speech, rather than an inability to orient attention to novel speech sounds. The authors presented high-functioning children with autism with two modifications of the oddball paradigm. In the first case, the speech non-unique “novels” (vowel /i/) were presented among the stream of non-speech standard and deviant stimuli. In the second case, the complex non-unique “novel” tone was imbedded among the standard and deviant speech sounds (vowels /a/ and /i/). Importantly, the “novels” in the study of Whitehouse and Bishop (2008) were not unique, i.e., they were repeated many times and therefore probably did not pop-out of the stimulation stream to the same degree as the unique and highly perceptually different stimuli applied by Courchesne et al. (1984) and Kemner et al. (1995). In different trials, the subjects were asked either to ignore the stimuli (passive condition) or to respond to the deviants (active conditions). P3a was measured as a difference between novel and standard stimuli. Under passive condition, P3a in ASD children was abnormally *reduced* in response to tone novels presented in the speech stream, but it was abnormally *increased* in response to speech novels presented among non-speech stimuli (both *p*'s < 0.05). No deficit, however, was found in the “active oddball” versions of the task when children attended to the stimuli. The authors concluded that the children with high-functioning autism were able to allocate attention to a novel speech sound if it was embedded in a sequence of non-speech auditory stimuli, but used top-down inhibition to attenuate responses to repeated streams of speech. Whatever the neural mechanisms underlying reduced processing of unattended stimuli in ASD are, the results of Whitehouse and Bishop clearly demonstrate the critical role of sustained attention to the sensory stream in the reported P3a findings in ASD. They also show that since the P3a abnormalities in ASD strongly depend on the second-order cognitive factors, they can hardly represent the primary deficit.

The role of attention in hypo- vs. hypersensitivity to change in ASD is further supported by functional Magnetic Resonance Imaging (fMRI) findings of Gomot et al. (2006, 2008). In the first study (Gomot et al., 2006), children were presented with a passive auditory novelty oddball task while watching a video. The participants with high-functioning autism activated “novelty detection network” in response to the novel stimuli significantly less than their age- and IQ-matched peers, and the maximal group difference was seen in the TPJ region. In a later study using the same stimuli (Gomot et al., 2008), researches asked participants to respond by a button press as soon as they heard a “strange” novel sound. In this active paradigm the children with high-functioning autism activated a few cortical regions, including inferior parietal area near TPJ, significantly greater than the control children. These fMRI findings in ASD were accompanied by shortened reaction times, showing that the higher brain activation was adaptive and advantageous for their behavioral response.

To sum up, the ERP/ERF studies focusing on two sequential stages of the brain response to the novel or deviant stimuli—automatic detection of physical changes indexed by MMN and evaluation of stimulus novelty indexed by the P3a component—found in individuals with ASD either increased, decreased or normal processing. These results suggest that individuals with ASD are generally able to detect changes in the stimulation stream when this stream is in the focus of their attention. Prominent problems arise when the deviant or novel stimuli appear in the presence of a strongly interfering task or stimulation (i.e., outside focus of attention). In this case, individuals with ASD may lack P3a observed in non-ASD comparison groups and demonstrate reduced amplitude of MMN. The fact that presence and direction of the MMN and P3a abnormalities in ASD depend on the context of stimulus presentation suggests that they do not reflect the primary deficits, but are rather preceded by a failure of even earlier triggering processes.

### Early pre-attentive arousal in sensory gating paradigm

The “sensory gating” paradigm that is specifically focused on pre-attentive arousal stages of auditory processing usually investigates “obligatory” ERP components in response to clicks. The pairs of clicks (“S1” and “S2”) separated by a short within-pair interval are presented with much longer inter-pair intervals. In adults, the so-called “obligatory” component P1 (also called P50), with latency of 50–80 ms, and N1, with latency of approximately 100 ms, decrease in amplitude with stimulus repetition, reflecting dampened processing of repetitive auditory input (“gating-out”). Usually, this inhibitory gating-out process is measured by a P50 S1/S2 amplitude ratio (but see also Kisley et al., 2004). The larger response to S1, on the other hand, reflects bottom-up arousal (Skinner et al., 2004) and/or initial orienting response (Atienza et al., 2001) caused by a rare and poorly predictable S1 sound, i.e., “gating in” (Boutros and Belger, 1999; Brenner et al., 2009).

The P50 component of AEP reflects ascending activation of the cholinergic arm of the reticular activation system (RAS) and is closely linked to pre-attentive arousal processes (Skinner et al., 2004). According to Skinner there are a few properties of P50 that support its association with RAS activity. Firstly, P50 is present during waking and rapid eye movement (REM) sleep, but is dampened during deep slow wave sleep (i.e., it is present when the cortex is activated). Secondly, it is mediated, at least in part, by cholinergic branch of arousal system. There is evidence for involvement of nicotine receptor-mediated cholinergic activity in the generation of P50 response to the S1 (Adler et al., 1999; Leonard et al., 2002). Thirdly, it rapidly habituates at stimulation rates greater than 2 Hz (i.e., it is sensitive to the “temporal novelty”) (Buchwald et al., 1992) and is sensitive to changes in stimulation (Nakagawa et al., 2013). Its generation in secondary auditory areas that receive input from non-lemniscal thalamo-cortical pathways (Howard et al., 2000) is also in line with its close involvement in arousal processes (Skinner et al., 2004).

**Figure 1:**
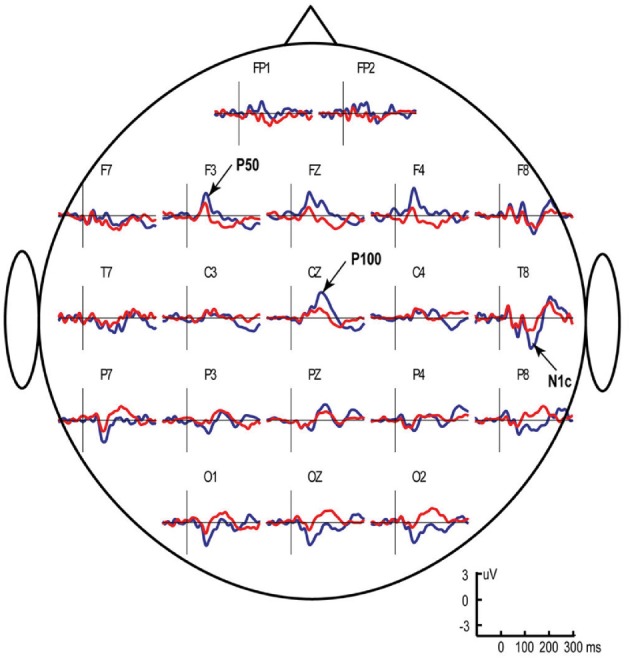
**Grand average ERP responses to left monaural “S1” (blue line) and “S2” (red line) clicks in typically developing children aged 3–8 years**. The S1–S2 interval was fixed at 1 s, while the S2–S1 interval varied between 7 and 9 s. Vertical lines mark stimulus onset. Note presence of two anteriorly positive components that comprise the “P1-complex”—P50 at approximately 70 ms after stimulus onset (maximal at frontal areas) and P100 at approximately 130 ms (maximal at Cz). The figure is adapted from Stroganova et al. (2013).

**Figure 2:**
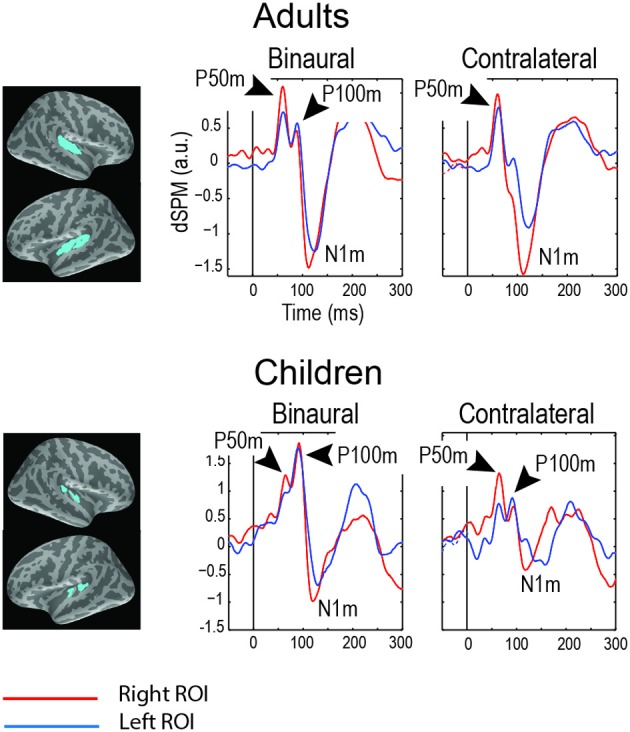
**Grand average magnetic auditory responses to binaural and monaural “S1” clicks in “neuro-typical” adults and children (8–15 years)**. The S1 click was presented after a long and variable interval (7–10 s) and was followed by the S2 click after 1 s. MEG source-specific dSPM time courses are given for the superior temporal regions (blue labels, see Orekhova et al., 2013, Figure 6 for details). The negative sign signifies incoming current and the positive sign signifies outgoing current. Note two distinct positive peaks of activity preceding N100m: P50m and P100m. The P50m is seen in both children and adults, while P100m is more prominent in children, especially in response to binaural clicks. The figure is adapted from Orekhova et al. (2013) with permission.

In children, the P50 (P50m in MEG) response to click is followed by another positive deflection of potential (Stroganova et al., 2013) (Figure 1) or magnetic field (Orekhova et al., 2013) (Figure 2) with latency of about 90–140 ms (P100 or P100m in MEG). The presence of the “P1 complex” comprising two positive waves (P50 and P100) thus distinguishes child response to the auditory clicks from the adult P50-N100 waveform. Similarly to that in P50, the strong attenuation of P100 with stimulus repetition suggests its link with arousal-like processes (Orekhova et al., 2012, 2013; Stroganova et al., 2013). The P100/P100m response to binaural or contralateral monaural S1 click is normally of higher amplitude in the right hemisphere. Such rightward lateralization may, at least partly, reflect greater involvement of the right hemisphere in sound localization, arousal, and attention processes (Hadlington et al., 2004; Ofek and Pratt, 2004; Howard and Poeppel, 2009; Nakagawa et al., 2013; Teshiba et al., 2013), although anatomical factors may also play a role (Shaw et al., 2013).

The vertex-positive P1 is thought to originate from extra-lemniscal projections to cortical pyramidal neurons in lower layer III and layer IV of the belt and parabelt areas of the auditory cortex (Eggermont and Ponton, 2003) and reflect bottom-up non-specific modulation of these areas (Kral and Eggermont, 2007). During childhood and adolescence the later part of the P1 complex (mainly represented by P100) is gradually canceled out in the surface EEG by the later maturing N100 component (Ponton et al., 2002; Eggermont and Ponton, 2003). In adult studies these waves are usually not separated and are therefore addressed to as P1 or P50. Even in adults, however, some researchers identified two positive waves contributing to P1, which were called P1a and P1b (Yvert et al., 2001). Conceivably, the adult P1a and P1b components correspond to the child's P50 and P100. In children the whole positivity between 50–140 ms is often mistakenly considered to be a developmental analog of the adult P50. However, the distinction between P50 and P100 in children is important, because these components may reflect different processes and, as we discuss later, are differently affected in ASD.

The sensory gating studies typically do not control for effects of direction of the subject's attention. It has been shown, however, that directing attention to the auditory stimuli does result in increase of the P1/S1 amplitude (White and Yee, 2006; Yee et al., 2010; Gjini et al., 2011). Therefore, if the effect of re-orienting is the main focus of interest, one should avoid directing subject's attention toward the auditory stimuli.

All published “sensory gating” studies in children with ASD employed silent movies to keep children busy and calm during auditory stimuli presentation. The stimuli were thus presented beyond the focus of child's attention. In case of the auditory P50, both S1 amplitude and S2/S1 ratio appear to be normal in non-retarded children with ASD (Kemner et al., 2002; Orekhova et al., 2008; Oranje et al., 2013). The decreased P50 S2/S1 ratio has been found only in those children with ASD who were mentally retarded (Orekhova et al., 2008). Even the retarded children, however, had normal P50 S1 amplitude. The only available adult study that applied “sound counting” modification of the paradigm has also reported normal S1 P50 amplitude and sensory gating in ASD participants (Magnee et al., 2009). These P50 findings sharply differentiate between individuals with ASD and those with schizophrenia who typically demonstrate P50 abnormalities, such as elevated S2/S1 ratio and decreased P50/S1 response (Chang et al., 2011).

Using a modification of the auditory sensory gating paradigm, Orekhova et al. (2012) have shown that despite having a normal P50, the 8- to 15-year-old children with ASD lacked normal rightward lateralization of the magnetic P100m response to binaural clicks. This abnormal lateralization was mainly due to the right-hemispheric P100m reduction in ASD and correlated with the presence of sensory modulation difficulties in auditory domain. A later EEG study applying a similar paradigm but using monaural clicks in younger (4- to 8-year-old) participants with ASD revealed reduction of the P100 S1 amplitude and P100 S2/S1 amplitude ratio to the left click “addressed” to the right hemisphere, but a fairly normal responses to the right clicks addresses to the left hemisphere. The reduced contralateral (right hemispheric) P100 response to the left click and the reduced rightward P100 lateralization in children with ASD correlated both with the presence of auditory modulation difficulties and with developmental delay. The right-hemispheric reduction of the auditory response to temporally novel (S1) binaural clicks in 4- to 8-year-old children with autism has been also found for a somewhat later (approximately 140 ms) obligatory ERP component N1c (Orekhova et al., 2009). Notably, similarly to P100, N1c to temporally novel stimuli was lateralized to the right hemisphere in the typically developing children, but not in those with ASD.

Collectively, these “sensory gating” studies consistently show that the processing of potentially salient but unattended and poorly predictable (presented with long and variable intervals) auditory stimuli is impaired in children with ASD. This impairment manifests itself in dampened reactivity of the right hemisphere at around 100 ms after stimulus onset and reflects the reduced bottom-up non-specific modulation of cortical auditory areas by reticular ascending arousal pathways. This right-hemispheric deficit in pre-attentive arousal may reflect the same neural deficit that underlies occasional behavioral unresponsiveness to auditory stimuli and even to stimuli of other sensory modalities in individuals with ASD and may contribute to their attention re-orienting difficulties.

The lateralized deficit in early pre-attentive arousal may result in a failure to activate the normally rightward-lateralized ventral attention network subserving attention re-orienting (Corbetta et al., 2008). This hypothesis predicts the presence of lateralized abnormalities in attention re-orienting behavior in people with ASD.

## Lateralized abnormalities in attention re-orienting in ASD

The brain network responsible for vigilance and attention re-orienting is strongly lateralized to the right hemisphere. Therefore, the unilateral damage to the right hemisphere, as compared to the unilateral damage to the left hemisphere, results much more frequently in decreased attentiveness to the contra-lateral left hemi-space (hemi-spatial neglect) and generally decreased arousal level (Mesulam, 1981; Robinson, 1985; Corbetta and Shulman, 2011).

Notably, left-sided unilateral neglect in patients with right brain damage is causally linked to both decreased arousal and its electrophysiological index—an auditory P1 response to binaural S1 clicks in the S1–S2 paradigm (Woods et al., 2012). Two available ERP studies in neglect patients that used monaural auditory stimuli showed reduction of the “obligatory” ERP responses to the left-sided as compared to the right-sided auditory stimulation (Deouell et al., 2000; Tarkka et al., 2011). This asymmetrical response attenuation in neglect patients resembles our finding in children with ASD suggesting similar neural deficiencies in the two clinical populations. Hence, it is likely that the right-hemispheric arousal deficit found in children with ASD may also lead to the left-sided attention deficit similar to that in neglect patients.

Two decades ago Bryson and Wainwright-Sharp hypothesized that attentional abnormalities in people with autism may represent a “developmental neglect syndrome” (Bryson et al., 1990). In line with this hypothesis Casey and colleagues have found that autistic savants had a particular difficulty with disengaging and shifting attention to the left hemispace (Casey et al., 1993). Although disengagement deficit in ASD individuals has been subsequently replicated in many studies across their life span (see Ames and Fletcher-Watson, 2010 for review), its dependence on the visual hemi-field was either not studied or was shown to be bilateral (Townsend et al., 1996).

It would appear that the above findings are at odds with our prediction that attention re-orienting difficulties in ASD are skewed to the left hemispace. However, even in patients with brain lesions and a sub-clinical form of the left-sided unilateral neglect, the left-sided extinction is evident only during high attention load at a fixation point (Bonato, 2012). Hence, such left-sided predominance of the attention disengagement deficit in individuals with ASD is likely to be subtle and may therefore critically depend on preceding engagement. Consequently, specific attributes of the experimental task applied to uncover symptoms of behavioral neglect in ASD may be of particular importance. It is interesting in this respect that, while performing a demanding spatial working memory task, the 3- to 5-year-old children with ASD experienced significantly greater difficulties when required to re-orient attention to the left rather than to the right from the previously attended location (Tsetlin et al., 2008). This right-sided bias was not observed in the typically developing age-matched control children. The other example of atypical attention lateralization in children with ASD comes from the face processing studies. While presented with a face, typically developing infants and children tend to make the first saccade to the left, while this normal left gaze bias is absent in young children with ASD (Guillon et al., 2013). Although absence of the left gaze bias may reflect face processing atypicalities in ASD, it is also generally in line with atypical lateralization of attention.

Kawakubo et al. (2007) provided the first electrophysiological evidence for dysfunction of the brain attentional disengagement system in autism. They found abnormal pre-saccadic potential in adults with autism during performance of a “gap-overlap” task requiring gaze shifts to peripheral targets. The atypically high pre-saccadic positivity in subjects with autism was found only under the “overlap” condition and has been assumed by the authors to reflect the allocation of extra effort for attentional disengagement. Interestingly, the authors reported a significant ANOVA Side × Condition interaction effect, which is illustrated in their Figure 3. Their results show that enhanced pre-saccadic positivity in subjects with autism preceded only *left* peripheral visual targets. This finding suggests that subjects with autism allocated more resources to divert their gaze to the left then to the right peripheral stimuli.

Apart from experiencing difficulties with orienting to the left hemispace, the neglect patients with right brain damage demonstrate abnormally speeded saccades to the right visual field (Natale et al., 2007). This rightward bias is thought to result from hyperexcitability of intact left hemispheric cortex due to its reduced inhibition by the damaged right hemisphere (Koch et al., 2008, 2013). Interestingly, abnormally speeded saccades to the right have been reported also in adults with autism (D'Cruz et al., 2009), thus indirectly supporting the hypothesis about the right-hemispheric deficit.

Collectively, the results of the above-summarized studies suggest some similarity between individuals with ASD and neglect patients with the right-hemispheric damage. Although to a different degree, both groups experience attention orienting bias and/or relatively greater difficulty in re-orienting to the left space, which is more evident when attention is strongly engaged into a previously attended spatial location. We would like to stress that the suggested role of the right-lateralized dysfunction in attention re-orienting problems in ASD does not mean that ASD is the “right-hemispheric disorder.” Indeed, structural abnormalities of gray and white matter in ASD are observed in both hemispheres (e.g., Travers et al., 2012; Greimel et al., 2013). An atypical pattern of structural cortical lateralization in children with ASD (Herbert et al., 2005) also implies an atypical development of both hemispheres rather than a specific unilateral deficit. The abnormal lateralization of language function to the right hemisphere was frequently reported in ASD and correlated with more severe language impairment (Eyler et al., 2012; Lindell and Hudry, 2013). Thus, there is convincing evidence that autism is associated with atypical brain lateralization and specialization as well as abnormal functioning of both hemispheres. Nevertheless, taking into account normally rightward lateralization of attention re-orienting function in the brain, the right-hemispheric dysfunction seems to be of particular relevance for attention problems in ASD.

Unlike neglect patients with brain lesions, the lateralized bias in ASD is likely to be explained by more subtle dysfunctions that particularly adversely affect the right-hemispheric attention networks. The abnormal cholinergic arousal may be one such mechanisms contributing to both lateralized ERP abnormality and attention re-orienting difficulties in ASD.

## Cholinergic abnormalities in ASD: possible effects on ERP/ERFs and behavior

Cholinergic neuro-modulatory system is critically involved in regulation of attention re-orienting and arousal (Everitt and Robbins, 1997; Sarter and Bruno, 2000; Sarter et al., 2005). Therefore, there is a good reason to believe that the cholinergic abnormalities found in individuals with autism (Deutsch et al., 2010; Anand et al., 2011) contribute to their attention and arousal regulation problems.

There are two major groups of cholinergic neurons. The first group is located in the basal forebrain and is often referred to as the magnocellular basal forebrain cholinergic system (Mesulam et al., 1983a,b; Mesulam, 1995). Nucleus basalis of Meynert is a major nucleus of the basal forebrain that projects to cerebral cortex and amygdala, while vertical limb nucleus of the diagonal band of Broca is an important cholinergic nucleus that innervates hippocampus. The second group is located in the brainstem in the region of the pedunculopontine tegmental nucleus (PPTg) and laterodorsal pontine tegmentum. Brainstem cholinergic neurons principally innervate the thalamus.

Cholinergic projection aids the processing of stimuli at a cortical level by enhancing the impact of salient information via a mechanism, which produces increases in signal to noise ratio (Robbins, 1997). The arousal-induced attentional processing (i.e., stimulus detection, selection and processing as a result of a novel, highly salient, aversive, or incentive stimuli) is mediated via the ability of bottom-up brainstem ascending noradrenergic projections to the basal forebrain to activate or “recruit” basal forebrain-telencephalic circuits (Sarter and Bruno, 2000). Thus, integrity of cholinergic projections of basal forebrain and their interaction with noradrenergic arousal is essential for efficient orienting of attention to novel and salient stimulation.

Remarkably, morphological abnormalities of basal forebrain were reported in children with autism. Riva et al. (2011) observed gray matter reduction in the basal forebrain of young children with ASD. The uncinate fasciculus (UF) is the major right-lateralized (Highley et al., 2002) fiber tract that connects inferior frontal and anterior temporal lobes and amygdale and carries cholinergic fibers from nucleus basalis of Meynert to these structures. Cheon et al. (2011) have found reduced fractional anisotropy and increased radial diffusivity in UF in boys with autism. This finding suggests reduced cholinergic modulation of fronto-temporal areas and amygdala. Neurons of another cholinergic branch located in diagonal band of Broca are also affected in ASD (Bauman and Kemper, 2005). Bauman and Kemper (2005) have shown that neurons of this nucleus were unusually large in the brains of children with autism younger than 13 years, whereas in brains of adult persons with autism (age 21 years and older) neurons were small, pale and decreased in number.

An interesting support for cholinergic system dysfunction in ASD comes from a recent study by Lemonnier et al. (2013). The authors have shown that children with ASD had significantly higher rate of “red dermographism”—a skin reaction involving the cholinergic system—than children exhibiting typical development. Yet another support comes from a sleep study that has found strongly reduced percentage of REM sleep in children with ASD compared with either typically developing or mentally retarded children without autism (Buckley et al., 2010). As acetylcholine is the main driver of REM sleep, Buckley et al. (2010) suggested that reduced REM/slow sleep ratio in autism may reflect a cholinergic dysfunction.

In the brain, acetylcholine acts through two major types of receptors—muscarinic and nicotinic. Reduced expression of cholinergic receptors has been reported in post-mortem brain tissues of people with autism (Perry et al., 2001; Martin-Ruiz et al., 2004; Ray et al., 2005) and was especially strong for α4 and β2 nicotinic acetylcholine receptor (nAChR) subunits in the “nonspecific” thalamic nuclei (Anand et al., 2011). Interestingly, strongly reduced concentration of the α4β 2-nAChR was found in persons with ASD in Brodman area 39, which overlaps TPJ—the cortical area principally involved in attention re-orienting (Martin-Ruiz et al., 2004). The nAChR abnormalities in ASD are likely to be post-transcriptional (Anand et al., 2011). Indeed, significant reduction in expression of the nAChR subunits, but not their mRNA, has been observed in post-mortem brains in autism (Anand et al., 2011). Neurorexin-1 abnormalities, reported in some individuals with autism, may also contribute to the cholinergic dysfunction because they result in abnormal targeting of α4β 2-nAChR to pre-synaptic terminals in neurons (Cheng et al., 2009).

Taking into account the convincing evidance about nAChR-mediated cholinergic disturbances in individuals with ASD it has been proposed that agonists and partial agonists for nicotinic acetylcholine receptors and modulators that enhance efficiency of these receptors may be useful for pharmacological treatment of autism (Deutsch et al., 2010; Anand et al., 2011). Preliminary clinical trials have indeed shown positive results (Nicolson et al., 2006).

There is evidence that the nAChRs are of particular importance for regulation of attention disengagement and shifting in neuro-typical individuals (Witte et al., 1997; Greenwood et al., 2005, 2009, 2012), while muscarinic receptors may underly tonic aspects of vigilance (Greenwood et al., 2009). Normal genetic variations in α4 subunit of nAChR have an impact on individual's ability to re-orient (disengage) attention. Individuals carrying two “T” alleles of the nAChR-α4 gen (T/T homozygotes) had the greatest cost of invalid relative to neutral cues in Posner's attention task, while C/C homozigotes demonstrated the greatest benefit of a valid cue (Parasuraman et al., 2005). The C/C homozygotes also had a greater ability to adapt (scale) their attention focus (Greenwood et al., 2005). It has been shown that the presence of different nAChR-α4 alleles affects brain activity during attention re-orienting, resulting in activation of different brain regions of the right temporo-parietal cortex (Giessing et al., 2012). These observations not only support involvement of nAChR-α4 in attention re-orienting in neurotypical individuals, but also provide a link between nAChRs abnormalities and attention re-orienting difficulties in ASD. Specifically, the nAChR-mediated deficits found in individuals with ASD may lead to pre-attentive arousal abnormalities and altered ERP/ERF responses to novel or salient stimuli.

It has been long known that cholinergic arousal mechanisms are involved in generation of P1 (P50) response to clicks in human adults and in animals (Buchwald et al., 1991; Skinner et al., 2004). Many studies linked the decreased P50 sensory gating in patients with schizophrenia with a particular type of nAChR—nAChR-α7 (Adler et al., 1999; Leonard et al., 2002; Martin and Freedman, 2007; Ishikawa and Hashimoto, 2011). The presence of a normal P50 in high-functioning people with ASD suggests a lack of considerable functional deficit of the nAChR-α7, at least in the brain circuits involved in P50 generation. The dampening of the later child-specific P100 component (Orekhova et al., 2012; Stroganova et al., 2013), on the other hand, could be potentially explained by functional deficit of another nAChR subtypes. One possible candidate is α4β 2-nAChR. Indeed, a post-mortem study has shown decreased expression of α4 and β 2 nA ChR subunits, but a normal α7 expression in parietal cortical areas of individuals with ASD (Martin-Ruiz et al., 2004). Possible role of nAChR-α4β 2 in P100 generation is also indirectly supported by the animal studies. These studies have shown that modulation of α4β 2-nAChR activation in mice does not influence the magnitude of hippocampal P20—the human analog of P50—but affects the amplitude of the component succeeding P20 (hippocampal N40) (Rudnick et al., 2009; Featherstone et al., 2012).

There is convincing evidence in the literature that nAChR stimulation mainly affects the attention reorienting network of the right hemisphere. Witte and colleagues observed that, in both humans and Rhesus monkeys, nicotine improved disengagement mainly by reducing reaction times to the targets presented in the left visual field (Witte et al., 1997). The authors therefore concluded that nicotine speeds processing mainly in the right hemisphere. Vossel et al. (2010) investigated effect of nicotine on attention in neglect patients and have shown that the drug improved re-orienting only in those patients in whom the right parietal and temporal cortex was intact (without lesions) and only in the case of re-orienting to the left hemi-space. The evidence for a possible asymmetrical effect of nicotine on obligatory ERP components comes from the study of Impey et al. (2013). They have found that acute nicotine administration increases the amplitude of visual P1 response to peripheral targets specifically in the right hemisphere of healthy individuals. Using fMRI, Thiel and Fink (2007) have found that during attention to visual and auditory stimuli nicotine modulated activity in multiple cortical regions, but that the only commonly affected area for the two modalities was the right superior temporal gyrus (right TPJ)—the crucial part of the ventral attention network.

To summarize, the nAChR-mediated arousal is critically involved in attention disengagement to peripheral targets and has a “left hemispace bias.” If stimulation of nicotinic receptors by nicotine primarily affects re-orienting primarily through activation of the right hemisphere, then putative nicotinic cholinergic dysfunction in ASD should reduce the right hemispheric response to the novel or salient unattended stimuli. In this way, nicotinic cholinergic dysfunction could explain reviewed findings on both right hemispheric preponderance in P100 abnormalities and left-sided bias in attention disengagement difficulties in ASD.

## Conclusions

Detection of new events occurring outside the focus of attention is fundamental to adaptive functioning and is most critical when attention is focused elsewhere. The unattended novel sensory events may demand further analysis according to their task relevance and may appear important for survival. Behavioral and physiological findings reviewed in this article imply that brains of many people with autism are, to a certain extent, impenetrable to such unattended but potentially salient changes in the immediate sensory environment. This deficit may lead to a spectrum of unusual behaviors that are typically observed in individuals with ASD and, being considered from different perspectives, appear as arousal regulation problems, attention re-orienting difficulties or abnormal modulation of the behavioral response to sensory events. Here we reviewed studies that applied ERP/ERF to investigate neural processing of salient (rare, novel, or deviant) auditory stimuli in ASD. We put forward a hypothesis that atypical processing of deviance and novelty in individuals with ASD may be grounded in the failure of nicotinic cholinergic arousal pathways to engage cortical mechanisms involved in detection of changes in the environment and appraisal of their novelty, if these changes occur beyond the currently attended sensory stream.

We hypothesize that in children with ASD the nicotinic cholinergic deficit, well documented in these disorders, manifests itself in reduction and abnormal lateralization of the *child-specific P100* ERP/ERF response to temporally novel acoustic events. The other stages of the brain response to a novel event—automatic detection of physical changes in auditory stream indexed by MMN and evaluation of stimulus novelty indexed by the P3a component—are strongly modulated in people with ASD by direction of their attention. Abnormal reduction of these components occurs only when the stimuli are presented beyond the attended auditory stream. This may reflect a failure of the earlier “cholinergic-arousal” processes to initiate attention re-orienting. The reduced cholinergic arousal thus appears to be a core neuro-functional deficit underlying slow and inefficient attention *re*-orienting in individuals with ASD throughout the life span.

The suggested link between the P100 abnormalities, the reduced nicotinic receptor-mediated activity, and the slow attention re-orienting in children with ASD allows formulating a few testable predictions for future research. *First*, it predicts that the child P100 component would be modulated by genetic variations of nAChR subunits in both typically developing children and in those with ASD. *Second*, taking into account the well-documented role of the nAChR-mediated arousal in attention re-orienting to the left hemispace, it predicts correlation between the right hemispheric P100 abnormalities and the lateralized attention re-orienting difficulties in ASD. We anticipate that in case of high attention load this re-orienting deficit should be more pronounced for left-sided stimuli. *Third*, the reduced nicotinic receptor-mediated activity may be present early in life and influence both P100 and attention re-orienting behavior in those infants “at risk” who would be later diagnosed with ASD. Further studies linking ERP/ERF findings with attention behavior and those searching for their neurochemical and genetic bases will help to understand causes of attention problems and sensory modulation difficulties in children with ASD and may prove helpful to direct early intervention.

## Author contributions

The authors contributed equally to the work, such as the conception and design of the paper, review of the literature, work drafting, and final approval of the version to be published. The authors agreed on all aspects of the work in ensuring that questions related to the accuracy and integrity of the work are appropriately investigated and resolved.

### Conflict of interest statement

The authors declare that the research was conducted in the absence of any commercial or financial relationships that could be construed as a potential conflict of interest.
